# Identification of *STRBP* as a Novel *JAK2* Fusion Partner Gene in a Young Adult With Philadelphia Chromosome-Like B-Lymphoblastic Leukemia

**DOI:** 10.3389/fonc.2020.611467

**Published:** 2021-01-11

**Authors:** Xin-Yue Zhang, Hai-Ping Dai, Zheng Li, Jia Yin, Xing-Ping Lang, Chun-Xiao Yang, Sheng Xiao, Ming-Qing Zhu, Dan-Dan Liu, Hong Liu, Hong-Jie Shen, De-Pei Wu, Xiao-Wen Tang

**Affiliations:** ^1^ National Clinical Research Center for Hematologic Diseases, Jiangsu Institute of Hematology, The First Affiliated Hospital of Soochow University, Suzhou, China; ^2^ Institute of Blood and Marrow Transplantation, Collaborative Innovation Center of Hematology, Soochow University, Suzhou, China; ^3^ Sano Suzhou Precision Medicine Co. Ltd., Suzhou, China; ^4^ Department of Pathology, Harvard Medical School, Brigham and Women’s Hospital, Boston, MA, United States

**Keywords:** fusion gene, *STRBP-JAK2*, Ph-like, chimeric antigen receptor, B-lymphoblastic leukemia

## Abstract

Philadelphia chromosome-like B-lymphoblastic leukemia (Ph-like ALL) describes a group of genetically heterogeneous, Ph-negative entities with high relapse rates and poor prognoses. A Janus-kinase-2 (*JAK*2*)* rearrangement has been reported in approximately 7% of Ph-like ALL patients whose therapeutic responses to JAK inhibitors have been studied in clinical trials. Here, we report a novel *STRBP-JAK2* fusion gene in a 21-year-old woman with Ph-like ALL. Although a normal karyotype was observed, a hitherto unreported *JAK2* rearrangement was detected cytogenetically. *STRBP-JAK2* fusion was identified by RNA sequencing and validated by Sanger sequencing. The Ph-like ALL proved refractory to traditional induction chemotherapy combined with ruxolitinib. The patient consented to infusion of autologous chimeric antigen receptor (CAR) T cells against both CD19 and CD22, which induced morphologic remission. Haplo-identical stem cell transplantation was then performed; however, she suffered relapse at just one month after transplantation. The patient subsequently received donor lymphocyte infusion after which she achieved and maintained a minimal residual disease negative remission. However, she succumbed to grade IV graft-*versus*-host disease 7 months post-transplant. In conclusion, this report describes a novel *STRBP-JAK2* gene fusion in a Ph-like ALL patient with a very aggressive disease course, which proved resistant to chemotherapy combined with ruxolitinib but sensitive to immunotherapy. Our study suggests that CAR T-cell therapy may be a viable option for this type of leukemia.

## Introduction

Philadelphia chromosome-like B-lymphoblastic leukemia (Ph-like ALL) is characterized by a gene expression profile resembling Ph-positive ALL (Ph+ ALL) despite an absence of *BCR-ABL1* rearrangement. Clinically, Ph-like ALL is deemed a poor-risk subtype of adult B-lymphoblastic leukemia due to the high possibility of induction failure, early relapse, and dismal outcome. Activation of the *JAK-STAT* pathway occurs in approximately 64% of Ph-like ALL patients, including 42% with *CRLF2* rearrangements and 22% with non-*CRLF2* rearrangements, both of which are therapeutically targeted by JAK inhibitors (1).

Chromosomal translocations, inversions, and deletions generating *JAK2* fusion genes and JAK-STAT activation occur in 7% of Ph-like ALL patients (2). Hitherto, 23 different partner genes are reportedly rearranged with *JAK2* in Ph-like ALL, including *ATF71P*, *BCR*, *EBF1*, *ETV6*, *OFD1*, *PAX5*, and *ZBTB46* (3). Most reports of these fusion genes have only presented genetic profiles at the expense of clinical findings. Hence, there are only limited data detailing the efficacy of JAK inhibitors and immunotherapy in patients with different *JAK2* rearrangements.

Here, we report a novel *STRBP-JAK2* fusion gene in a patient with Ph-like ALL which proved resistant to standard induction chemotherapy and ruxolitinib, but achieved complete remission (CR) after chimeric antigen receptor (CAR) T-cell therapy, allogenic stem cell transplantation (allo-HSCT) and donor lymphocyte infusion (DLI).

## Methods

### Fluorescence *In Situ* Hybridization

FISH analyses were performed according to our institutional protocols (4). Accordingly, a commercial panel of FISH probes covering Ph-like ALL, including *ABL1*, *ABL2*, *CRLF2*, *EPOR*, and *JAK2*, was purchased (Vysis, Abbott/IL/USA). A positive rearrangement was reported when at least 3% of the nuclei showed break-apart split signals. Bone marrow (BM) samples at diagnosis and at 28 days after CAR T-cell therapy were analyzed by FISH (Olympus IX 71, Tokyo, Japan).

### RNA Sequencing and Nested RT-PCR

Total RNA was extracted from diagnostic BM samples using RNeasy Mini Kit (Qiagen, Hilden/Germany). RNA-sequencing libraries were prepared with 20–100 ng total RNA with the TruSight RNA Fusion Panel (Illumina, San Diego/CA/USA). All reads were independently aligned with STAR (version 2.3) against the human reference genome. For nested RT-PCR, sequences of the first pair of primers specific to *STRBP* were: (5′-AGAAAGTGGCAAAGGCGAGT-3′); and to *JAK2* (5′-GCACATCTCCACACTCCCAA-3′). Primers for the second round of PCR specific to *STRBP* were: (5′-TTATCCCTCAGGCAAAGGGC-3′); and to *JAK2:* (5′-GGTCTTCAAAGGCACCAGAAAAC-3′).

## Definition

CR was defined according to the consensus criteria (5). Minimal residual disease (MRD)-negative remission was defined as less than 0.01% of marrow blasts by multiparameter flow cytometry (FCM).

## Results

**Figure 1:**
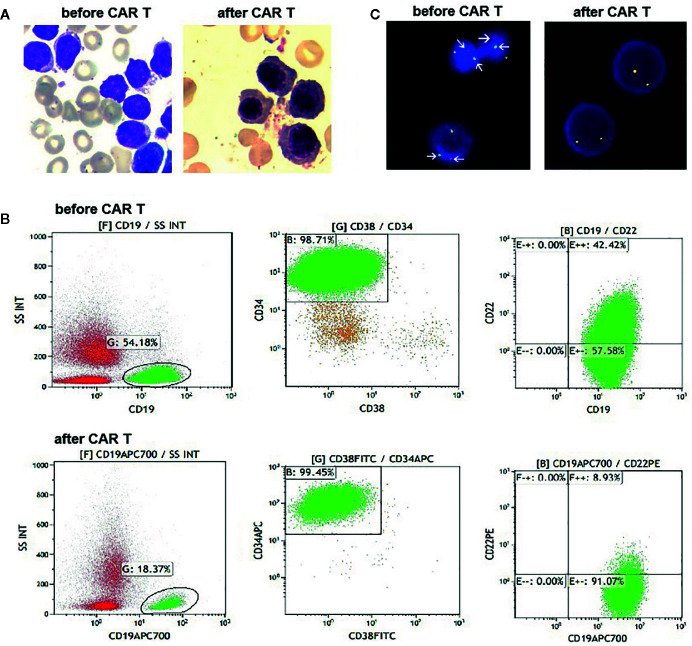
**(A)** Bone marrow (BM) aspirates before and after CAR T cells infusion. At day 28 post infusion, there was no evidence of blasts in the BM. **(B)** Flow cytometry (FCM) analysis of BM samples before and after CAR T cells infusion. **(C)** Fluorescence *in situ* hybridization results with a *JAK2* break-apart probe, which detected split signals of *JAK2* before infusion of CAR T cells (arrow), and normal JAK2 signals after infusion of CAR T cells.

A 21-year-old woman presented with petechiae in the lower extremities in February 2018. Her complete blood count showed a white blood count of 55.4 × 10^9^/L, hemoglobin 110 g/L, and platelets 18 × 10^9^/L. BM morphology analysis detected 88% blasts (**Figure 1A**). FCM detected 61.45% blasts, which were positive for CD10, CD19, CD22, CD33, CD34, cCD79a, and HLA-DR (**Figure 1B**). Conventional karyotyping was normal. Results of multiplex PCR covering 41 fusion genes commonly detected in acute leukemia were negative. Targeted next-generation sequencing covering 172 genes reportedly mutated in hematological malignancies revealed *NOTCH1* G881S and *BCOR2* R1589H mutations: both germline.

The patient was treated with conventional induction chemotherapy, including idarubicin, vincristine, pegaspargase, and dexamethasone. Ruxolitinib (20 mg, twice daily) was administered after *JAK2* rearrangement was detected by FISH. Unfortunately, BM analysis revealed 46.5% blasts at the end of the induction therapy. Accordingly, she was enrolled in the CAR T-cell clinical trial (NCT03614858). She received MVP (mitoxantrone 10 mg on day 1, vincristine 4 mg on day 1, and dexamethasone 14 mg on days 1–7) regimen to reduce her tumor burden, followed by lymphodepletion. Then, 1.5 × 10^7^/kg tandem CD19/CD22 CAR T cells were infused *via* a dose-escalation schedule for five consecutive days (0.5 × 10^6^/kg, 1.5 × 10^6^/kg, 3 × 10^6^/kg, 5 × 10^6^/kg and 5 × 10^6^/kg). She suffered grade 3 cytokine release syndrome, characterized by fever, hypotension and hypoxia. Symptoms of macrophage activated syndrome, including high fever, increase of ferritin, fibrinogen depletion and decreased NK cell activity, were also observed. All of the above complications were alleviated with dexamethasone (5 mg/d for 2 days), fluid boluses, vasopressors and supplemental oxygen. Typical symptoms of CAR T-cell related encephalopathy syndrome were not observed. Her changes of blood cell count and the persistence of the CAR T cells are shown in **Supplementary Figures 1A, B**. At day 28 after the CD19/CD22 CAR T-cell infusion, no blasts were detected on her BM smear (**Figure 1A**). However, 5.9% neoplastic cells were detected with FCM, which tested positive for CD10, CD19, CD33, CD34 but negative for CD22 (**Figure 1B**). She tested negative by FISH for *JAK2* rearrangement (**Figure 1C**). Haplo-identical allo-HSCT was performed one month after the CAR T-cell therapy. Cyclosporin (3 mg/kg/d), mycophenolate mofetil (30 mg/kg/d) and low dose methotrexate (15mg/m^2^ at d1, 10 mg/m^2^ at d3, d6, and d10) were administered to prevent graft-*versus*-host disease. Unfortunately, early hematological relapse was detected at 30 days after allo-HSCT. Cyclosporin and mycophenolate mofetil were withdrawn at day 30 and day 28, respectively. Because of persisting blasts in the BM, donor lymphocytes were infused at day 60 after the allo-HSCT. MRD negative remission was achieved after the DLI; however, she died of grade IV graft-*versus*-host disease involving the intestine and liver at 7 months post-HSCT.

**Figure 2:**
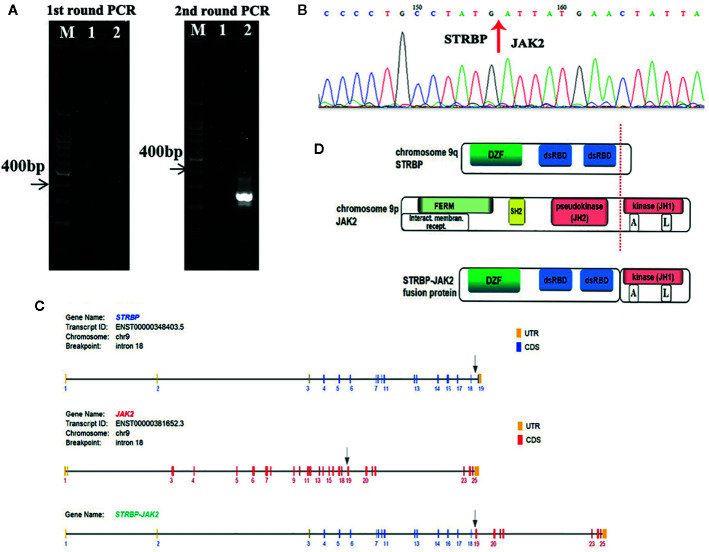
**(A)** Identification of the *STRBP-JAK2* fusion transcripts by nested RT-PCR. Lane M, DNA ladder; Lane 1, PCR negative control; Lane 2, *STRBP-JAK2*. **(B)** Sanger sequencing result, which confirmed a fusion between exon 18 of *STRBP* and exon 19 of *JAK2*. **(C)** Schematic representation of the predicted *STRBP-JAK2* fusion gene. **(D)** Schematic representation of the predicted STRBP-JAK2 fusion protein.

FISH analyses with split signal probes covering *ABL1*, *ABL2*, *CRLF2*, and *EPOR* all tested negative (data not shown). A split-signal of *JAK2* was detected in 68% interphase nuclei from BM samples (**Figure 1C**). However, BM samples taken 28 days after CAR T-cell therapy showed normal intact signals for *JAK2* (**Figure 1C**). RNA sequencing of diagnostic samples revealed in-frame fusion of exon 18 of *STRBP* with exon 19 of *JAK2*. The first round RT-PCR showed a fusion specific band, while the second round showed a sharp band, of a size (262bp) consistent with *STRBP-JAK2* fusion, which was absent in placental control RNA template (**Figure 2A**). Sanger sequencing of the PCR band confirmed that the *STRBP* exon 18 was fused to *JAK2* exon 19 **(** **Figures 2B, C**).

## Discussion

*JAK2* is located on chromosome 9p24, and encodes a non-receptor tyrosine kinase that participates in the activation of the JAK-STAT pathway and plays a central role in regulating cell proliferation, differentiation, survival, and apoptosis during hematopoiesis (6). *JAK2* contains an N-terminal FERM domain that is required for erythropoietin receptor association, a central Src homology 2 (SH2) domain that binds to STAT transcription factors, a pseudokinase domain (JH2) and a C-terminal tyrosine kinase domain (JH1) (**Figure 2D**). The JH2 domain can regulate activity of the JH1 domain. Deletion of the JH2 domain leads to enhance the tyrosine kinase activity of *JAK2* and increase phosphorylation of intracellular signaling molecules, including STATs, PI3K/AKT/mTOR, and MAPKs (7). A high proportion of patients with myeloproliferative disorders carry dominant gain-of-function V617F mutations in the JH2 domain, which lead to constitutive tyrosine phosphorylation activation and excessive cell proliferation (8). *JAK2* rearrangements such as *PCM1-JAK2* are frequently detected in myeloproliferative diseases with eosinophilia (9). *JAK2* rearrangements have also been observed in Ph-like ALL, chiefly chromosomal translocations resulting in fusion of *JAK2* with *EBF1*, *ETV6* and *OFD1*, or inversions resulting in fusion of *JAK2* with *PAX5* or *RFX3* (10–14). Consequently, *JAK2*-related fusion genes served as driver mutations of Ph-like ALL, by causing hyperactivation of the JAK-STAT signaling pathway, deregulated cell proliferation and resistance to apoptosis (7).

*STRBP*, the spermatid perinuclear RNA-binding protein, is located on chromosome 9q33 and has widespread expression in lymph nodes, testes, and other tissues. Structurally, it contains a domain associated with zinc fingers (DZF domain) and two double-stranded RNA-binding domains (dsRBD) (**Figure 2D**). It is a double-stranded RNA binding protein which also associates with microtubules, and, accordingly, plays an important role in mammalian spermatogenesis (15). Reports of rearrangements involving *STRBP* have been hitherto limited in solid tumors, *e.g. STRBP-ASTN2* observed in lung adenocarcinoma and *STRBP-ATG14* observed in breast cancer (16). No functional analyses of these two fusion genes have been described so far. Consequently, this is the first report of *STRBP* involvement in a hematological malignancy. Indeed, this is the first case of *STRBP-JAK2* fusion gene reported in Ph-like ALL. The *STRBP-JAK2* fusion contained the 5′ *STRBP*, including DZF and dsRBD, and the 3′ *JAK2* with an intact protein kinase domain (JH1) (**Figure 2D**). Because DZF and dsRBD domains of STRBP promote oligomerization in a wide variety of proteins, including STBRP itself (17), we propose an oncogenic model whereby *STRBP-JAK2* homodimerization, driven by the DZF and dsRBD domains from STRBP, leads to auto- and trans-phosphorylation within the activation loop of the JAK2-JH1 domain and, thereby, constitutive activate JAK-STAT signaling.

*JAK2* rearrangements universally result in an in-frame fusion of the carboxyl terminal portion of *JAK2* with its intact tyrosine kinase domain, joining in-frame to the amino terminal portion of the partner gene. Structurally, *STRBP-JAK2* fusion is congruent with previously reported fusion models (3). In this case, both *JAK2* and *STRBP* are located on chromosome 9, implying that *JAK2-STRBP* fusion may result from inversion of chromosome 9. Both inv(9)(p24q33) and t(9;9)(p24;q33) are cryptic rearrangements, and due to the dearth of good metaphases in BM samples at diagnosis, we were unable to distinguish between these options in the affected patient.

In Ph-like ALL, *JAK2* exhibits high levels of promiscuity, engaging with 23 different fusion partners reported in more than 50 patients hitherto. Most of these fusion genes were identified by RNA sequencing, but regrettably, little is known about their treatment responses and prognosis. Characteristics of 10 of these patients, where therapeutic details have been reported, are summarized in **Supplementary Table 1** (including the current case). In addition to *JAK2* fusion, five of the nine patients had concurrent deletions of *IKZF1*, which is consistent with the finding that an *IKZF1* deletion is the most common secondary aberration in Ph-like ALL (2). Four of the 10 patients received combined treatments with ruxolitinib. The patient with *GOLGA5-JAK2* achieved a CR with high dose ruxolitinib (40 mg/m^2^, bid) (18), while neither the patient with *RNPC3-JAK2* (19) nor the current one with *STRBP-JAK2* responded to ruxolitinib. In our patient, ruxolitinib was administered at the standard dosage used for treatment of myeloproliferative diseases (20 mg bid), and whether she would benefit from an increased dose remains unknown. Six patients were reported to have undergone allo-HSCT, without long-term follow-up data. Our patient achieved morphologic remission after CAR T-cell therapy and MRD negative remission after DLI in the setting of an early relapse after allo-HSCT, which implies that she benefited from immunotherapies.

In conclusion, we have characterized a novel *STRBP-JAK2* fusion gene for the first time in a refractory Ph-like ALL patient who failed to respond to conventional chemotherapy plus ruxolitinib. Our study suggested that the CAR T-cell therapy might, however, prove a viable option in this entity.

## Data Availability Statement

The original contributions presented in the study are included in the article/**Supplementary Material**. Further inquiries can be directed to the corresponding authors.

## Ethics Statement

The studies involving human participants were reviewed and approved by the First Affiliated Hospital of Soochow University. The patients/participants provided their written informed consent to participate in this study.

## Author Contributions

H-PD, ZL, JY, D-PW and X-WT treated the patients. X-PL, C-XY, SX, HL, and H-JS designed the genetic analysis and interpreted the data. D-DL performed the morphology analysis. M-QZ performed flow cytometry analysis. X-YZ and H-PD wrote the manuscript. D-PW and X-WT critically read and revised the manuscript. All authors approved the final version of the manuscript. All authors contributed to the article and approved the submitted version.

## Funding

This work was supported by research grants from the National Natural Science Foundation of China (81873443), National Science and Technology Major Project (2017ZX09304021), National Key R&D Program of China (2016YFC0902800), Priority Academic Program Development of Jiangsu Higher Education Institutions (PAPD), Frontier Clinical Technical Project of the Science and Technology Department of Jiangsu Province (BE2017655), Jiangsu Provincial Medical Talent (ZDRCA2016045), Major Natural Science Research Projects in institutions of higher education of Jiangsu Province (19KJA210002), The Key Science Research Project of Jiangsu Commission of Health (K2019022), National Natural Science Foundation of China (81700139), and National Science Fund of Jiangsu Province (BK20170360).

## Conflict of Interest

X-PL and C-XY are employed by the company Sano Suzhou Precision Medicine Co., Ltd.

The remaining authors declare that the research was conducted in the absence of any commercial or financial relationships that could be construed as a potential conflict of interest.
