# Supplementary figures and images for: Identification of STRBP as a Novel JAK2 Fusion Partner Gene in a Young Adult With Philadelphia Chromosome-Like B-Lymphoblastic Leukemia
Source: Front Oncol. 2021 Jan 11;10:611467. doi: 10.3389/fonc.2020.611467 (PMC7831028; doi:10.3389/fonc.2020.611467)

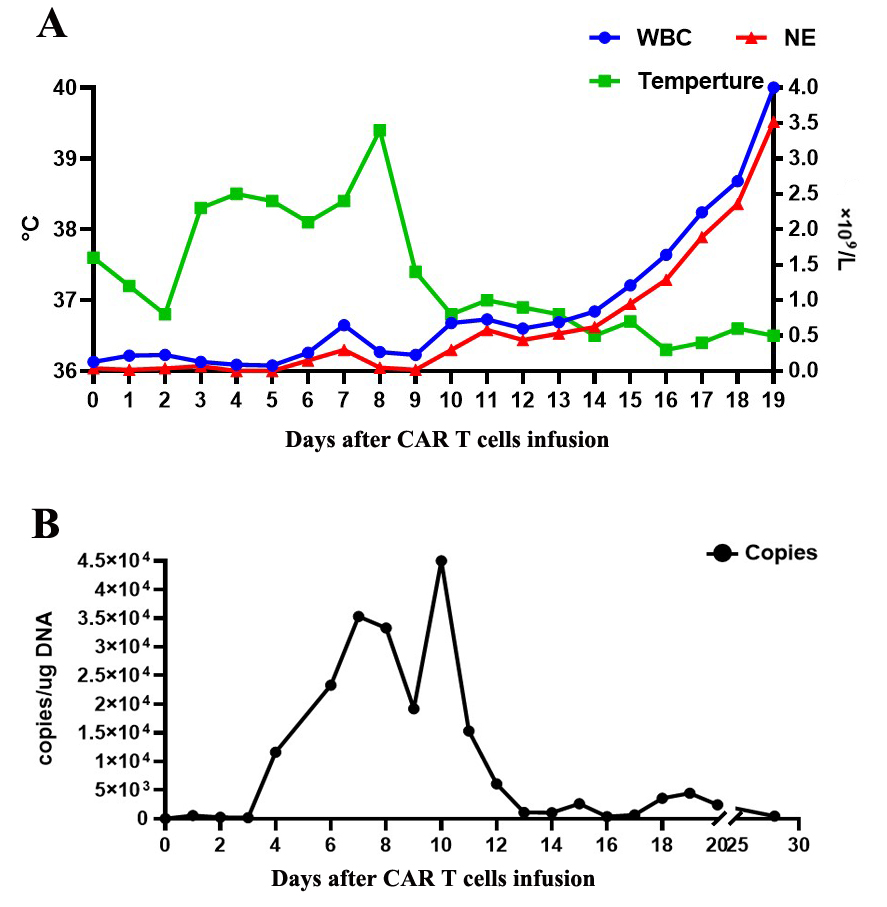

Supplement: Supplementary file 1 [file Image_1.jpeg]
