# Supplementary material for: Identification of STRBP as a Novel JAK2 Fusion Partner Gene in a Young Adult With Philadelphia Chromosome-Like B-Lymphoblastic Leukemia
Source: Front Oncol. 2021 Jan 11;10:611467. doi: 10.3389/fonc.2020.611467 (PMC7831028; doi:10.3389/fonc.2020.611467)
Supplement: Supplementary file 2 [file Table_1.doc]

**Supplementary Table 1. Clinical, karyotype and genetic features of 10 cases of acute B lymphoblastic leukemia with JAK2 rearrangements**

| **Patient**  **No.** | **Age/sex** | **Karyotype** | **Fusion partner** | **Other genes mutated** | **Target therapy** | **HSCT** | **Survival** | **Reference (DOI)** |
| --- | --- | --- | --- | --- | --- | --- | --- | --- |
| 1 | 2.7/M | 47, XY,+2, del(2)(p23), t(3;22;9) (p12;q11.2; p24) [10]/46, XY[2] | BCR | IKZF1 (IK6), EBF1, PAX5 CDKN2A/CDKN2B | N/A | N/A | unknown | [10.1016/j.ccr.2012.06.005](https://doi.org/10.1016/j.ccr.2012.06.005) |
| 2 | 10/M | Complex karyotype* | GOLGA5 | IKZF1 deletion | Ruxolitinib | Y | 20months post HSCT | [10.3324/haematol.2018.192088](https://dx.doi.org/10.3324/haematol.2018.192088) |
| 3 | 11/M | 46,XY,-17+mar 1[11]/46, XY[9] | OFD1 | None | N | Y | N/A | 10.1111/bjh.13757 |
| 4 | 12.9/F | 47, XX, r(7)(p12q31),+9[14]/46, XX[6] | PAX5 | IKZF1 deletion | N/A | N/A | unknown | [10.1016/j.ccr.2012.06.005](https://doi.org/10.1016/j.ccr.2012.06.005) |
| 5 | 29/F | 46,XX,t(1;9)(p13;p22) | RNPC3 | NRAS, PAX5 | Ruxolitinib | Y | 7months after diagnosis | [10.1002/mgg3.1110](https://doi.org/10.1002/mgg3.1110) |
| 6 | 14/M | 46,XY,t(9;17)(p24;q21)[17/20] | SPAG9 | None | N/A | Y | 7 months | 10.1002/gcc.22251 |
| 7 | 14/M | 46,XY,t(5;9)(q12;p1?3) | SSBP2 | None | Ruxolitinib | N | unknown | 10.1056/NEJMoa1403088 |
| 8 | 12.2/F | N/A | STRN3 | IKZF1 deletion | N/A | N/A | unknown | [10.1016/j.ccr.2012.06.005](https://doi.org/10.1016/j.ccr.2012.06.005) |
| 9 | 33/F | 46,XX,t(3;9)(q13;p24) | ZBTB20 | IKZF1 deletion | Blinatumomab | Y | 200days post HSCT | [10.1101/mcs.a004937](https://doi.org/10.1101/mcs.a004937) |
| 10 | 24/F | Normal karyotype | STRBP | NOTCH1 BCOR1 | Ruxolitinib | Y | 7months post HSCT | The present case |

*46,XY,der(6)t(6;14)(p23;q32)del(6)(q15q23),der(9)inv(9)(p?q?34)del(9)(p21p13)del(9)(q22q31),der(14)t(6;14)(p23;q32)[14].ish,der(6)(IGH+),der(14)(IGH-)/46,XY[4]
